# Technological Assessment of MEMS Alkali Vapor Cells for Atomic References

**DOI:** 10.3390/mi10010025

**Published:** 2018-12-31

**Authors:** Pawel Knapkiewicz

**Affiliations:** Faculty of Microsystem Electronics and Photonics, Wroclaw University of Science and Technology, Wroclaw 50-372, Poland; pawel.knapkiewicz@pwr.edu.pl

**Keywords:** MEMS cells, atomic references, frequency standards, microfabrication

## Abstract

This paper is a review that surveys work on the fabrication of miniature alkali vapor cells for miniature and chip-scale atomic clocks. Technology on microelectromechanical systems (MEMS) cells from the literature is described in detail. Special attention is paid to alkali atom introduction methods and sealing of the MEMS structure. Characteristics of each technology are collated and compared. The article’s rhetoric is guided by the proposed classification of MEMS cell fabrication methods and contains a historical outline of MEMS cell technology development.

## 1. Introduction

Atomic time and frequency standards based on the absorption of microwaves by Cs/Rb vapors were discovered in the 1950s [1,2]. They soon became the most accurate frequency references for time and frequency measurements, playing a very important role in today’s technology [3,4,5].

Miniaturization of atomic clocks became a hot topic in the late 1990s after a series of publications showed technical and phenomenological fundamentals of fully optical coherent population trapping (CPT) atomic clocks [6,7,8].

The first technical realizations of microelectromechanical systems (MEMS) miniaturized Cs/Rb atomic clock were achieved by Kitching [9,10]. Several conceptual works paved the way for obtaining a laboratory prototype of the chip-scale atomic clock (CSAC) [11,12,13,14,15,16,17,18,19,20,21,22,23,24,25,26,27,28,29,30]. Microsemi Corporation provides the CSAC SA.45s miniature atomic clock, which is the only commercially available clock of its type. In 2015, Seiko Epson Corporation revealed the AO6860LAN model CSAC [31], although detailed information about the solution, including technical and sales documentation, are not available.

The operational principles and the detailed description of CSAC have been widely shown in the literature relating to the subject and therefore will not be discussed here [3,6,32,33,34,35,36,37,38,39,40].

This article concentrates on the fabrication problem of one of the most important parts of CSAC, i.e., the MEMS Cs/Rb cell. The cell has to be small and hermetically sealed to protect the alkali vapors and buffer gases. The cell structure must allow light to pass to and from atoms. The material from which the cell is made cannot react with the atoms and cannot be magnetic. The wafer-scale fabrication, including the cell filling, should be possible [34].

The first realization of the miniature atomic clock cesium MEMS cells gave very promising results. However, not long afterward, it was noted that the parameters of the cells were more problematic than expected. The cells have shown significant instabilities of time parameters. Moreover, some of the very perspective and technological solutions and methods of cesium fabrication and rubidium MEMS cells for miniaturized atomic clocks have appeared to be a technical dead end.

The cell fabrication and alkali metal introduction methods have been reported in many articles (description and references will be given later). Other review works [35,41] contain published information. The main goal of this article is to review the methods of fabrication of MEMS cells for CSAC, with a discussion on their advantages and disadvantages. In contrast to the previously mentioned review works, a classification of the alkali vapor cell technologies is proposed, and each technology is described in detail. The newest technological concepts are also included in the discussion.

## 2. Methods of MEMS Vapor Cell Fabrication

**Figure 1:**
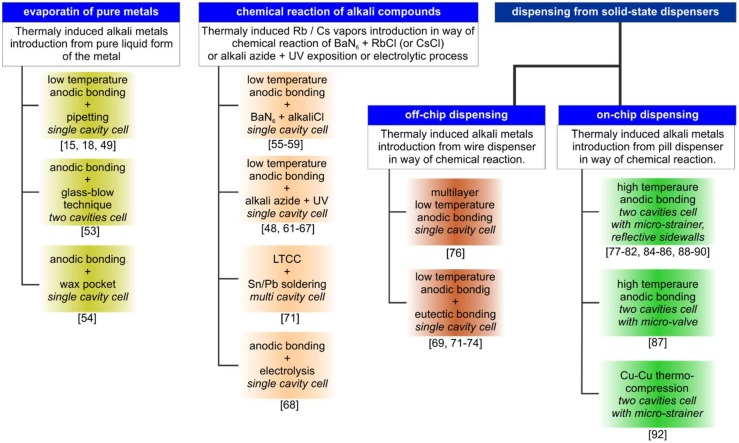
Fabrication “family tree” of alkali microelectromechanical systems (MEMS) cells.

Several methods of MEMS cell fabrication are applied in microfabrication techniques and inner atmosphere generation, including buffer gas and the introduction of alkali metal vapor. These methods are based on three main principles: evaporation of pure liquid alkali metals, chemical reaction of alkali chemical compounds, and dispensing of alkali vapor from solid-state dispensers (Figure 1).

Presented here are cells fabricated with MEMS technology, including silicon and glass micromachining (reactive-ion, deep reactive-ion, or potassium hydroxide etching), anodic bonding as the sealing process, or thin films deposition. The term “MEMS cell” is used intentionally as it fully reflects the fabrication methods, the small size of the cells, and the pursuit of mass and cheap production.

**Figure 2:**
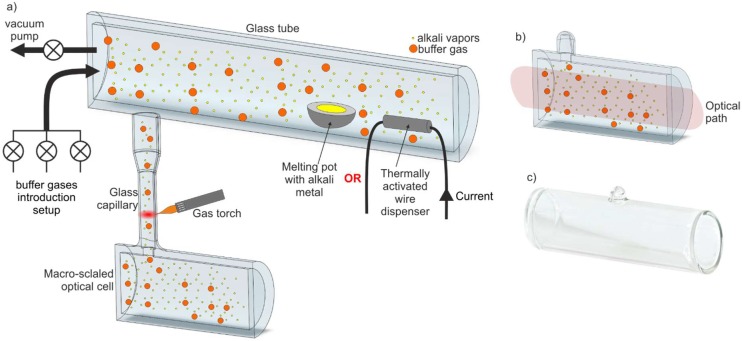
Set-up of the glassblowing technique, which allows fabrication of macroscale cells: (**a**) visualization of the set-up, (**b**) macroscale cell after cutting out, (**c**) an example of glass macroscale commercial cell.

Before presenting the fabrication methods of the integrated Cs/Rb optical version of microcells, the glassblowing technique, a well-known method for fabrication of mesoscale and macroscale glass alkali vapor cells, must first be presented. The basic configuration of the technical set-up applied in that technique is shown in Figure 2a.

The set-up is built on the basis of a standard vacuum apparatus equipped with a specialized glass section. The section consists of a glass tube connected to a vacuum pump and a gas introduction line. A melting pot with alkaline metal (historical first) or a wire dispenser is located inside the glass tube. The glass macroscale cell (Ø = 19 mm or 25.4 mm, length—75 mm or 100 mm) is connected to the glass tube through a glass capillary.

In the first step, all parts are pumped to about 10^−5^ Torr, which is then followed by the introduction of a buffer gas, or mixture of buffer gases, inside the glass tube and the cell. Cs/Rb vapor is delivered by the evaporation of pure liquid metal or by dispensing atomic Cs/Rb from a wire dispenser, with the latter of the two methods being seemingly more popular. Alkali vapor diffuses to the cell kept at ambient temperature and condenses inside the cell, forming liquid drops. Next, the connecting glass capillary is cut out using a gas torch, and the cell is separated from the glass tube (Figure 2b,c).

The glassblowing, meso/macro method is, as previously mentioned, commonly applied for fabrication of reference alkali cells. This technique also allows for the manufacturing of cells with antireflective and/or “antirelaxation” layers. There are plenty of manufactures offering reference cells made of quartz or borosilicate glass filled up with Cs or Rb atoms as well as with a composition of buffer gases made on request [42,43,44]. Even though the glassblowing technique yields excellent results and is widely used, it cannot be directly applied in MEMS-integrated alkali cell technology.

### 2.1. Introduction of Pure Alkali Metals

#### 2.1.1. Pipetting

**Figure 3:**
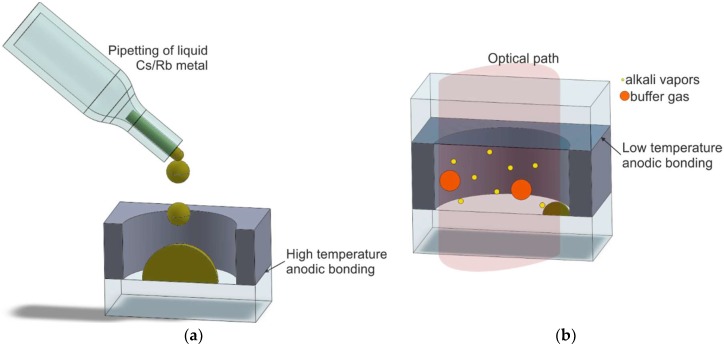
Fabrication process with direct alkali metal introduction utilizing pipetting: (**a**) liquid metal pipetting to silicon–glass preform, (**b**) cross section of the cell.

The fabrication of glass–silicon-integrated MEMS cesium vapor cell, which is historically the oldest process of its kind, was proposed by Kitching and his group [45]. The fabrication procedure consists of three main steps (Figure 3):

(1) fabrication of glass–silicon preform (deep reactive-ion etching, high-temperature anodic bonding),
(2) direct alkali metal introduction through pipetting, and
(3) final encapsulation by low-temperature, long-lasting anodic bonding.

The cell consists of deep reactive-ion (DRIE)-etched silicon body and two glass covers bonded anodically to the body. First, anodic bonding is performed under optimal conditions (temperature of about 400 °C, polarization of about 1 kV) (Figure 3a).

The final encapsulation is done inside a high-vacuum chamber (10^−5^ Torr or better). The preform is placed onto a hotplate at room temperature and filled with liquid cesium via pipetting. This is followed by buffer gas, introduced under absolute pressure between 50 and 200 Torr. Glass cover 2, previously distanced from the silicon–glass preform, is placed onto the preform, and low-temperature, long-lasting anodic bonding is done. First, the polarizing voltage is applied, and the temperature of the process is then elevated slowly to the desired level (below 350 °C). The process lasts several hours (≤24 h).

This procedure, in this case, produced good results at the preliminary stage of inventing a physical package of microatomic clocks [15,18,46], but several negative features of the proposed technology ruined the long-term properties of the cell. As is common knowledge, the optimal anodic bonding process needs a reasonably high process temperature ranging from 400 °C up to 450 °C. Bonding between silicon and glass is done by the formation of a siloxane bond (Si–O–Si groups), resulting from dehydration and dissociation of molecular water covering hydrophilically bondable surfaces. The resulting residual gas particles (O_2_, H_2_O, OH^−^, hydrocarbons) are reactive to alkali metals. Lowering the encapsulation temperature below 350 °C preserves alkali vapors against unwanted reactions with products of anodic bonding chemistry, but the bonded silicon–glass interface remains chemically unstable and generates oxidizing agents. This leads to significant deterioration of the alkali atom atmosphere and optical absorption properties of the cell. Detailed discussion of anodic bonding chemistry and optimization of the process is commonly accessible in the literature relating to the subject [47,48], but there is a common consensus that good anodic bonding needs high temperatures and sufficient time.

Losev et al. [49] proposed a glass cell fabrication method that utilized direct introduction of rubidium metal and glass fusion with CO_2_ laser. The technology is interesting, but it is based on precision mechanics rather than MEMS technology; therefore, this solution will not be discussed further in this review.

#### 2.1.2. Glassblowing in Microscale

**Figure 4:**
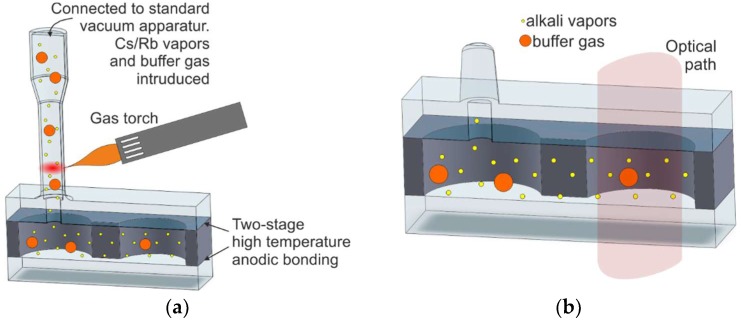
Hybrid process of glassblowing and microfabrication: (**a**) alkali vapor introduction to MEMS structure and cut off with gas torch, (**b**) cross section of the structure.

The hybrid process of glassblowing and microfabrication, presented in Reference [50], is a combination of the conventional vapor cell fabrication methods and specifics of microfabrication. The process starts with the etching of two via holes, 1.5-mm external diameter each, in a silicon wafer. The holes are connected by a 1-mm-wide and 100-µm-thick channel, made separately. In the next step, the top and bottom Pyrex wafers are attached via anodic bonding. The top wafer has a 0.5-mm via hole drilled above one of the holes (Figure 4a). The connecting pipe is attached to the vacuum line where alkali is dispensed, as described earlier (Figure 4b).

Next, the cell is filled with alkali metal and buffer gas, and the tabulation is cut off with a gas torch.

This method of fabrication presents all the advantages of the conventional glassblowing technique and ensures optimal, high-temperature anodic bonding of the glass and silicon parts of the cell, resulting in pure cesium vapor and extremely well-controlled composition and pressure of buffer gas. However, the process is not fully compatible with MEMS technology, so mass-scale production may prove problematic.

#### 2.1.3. Wax Pockets

**Figure 5:**
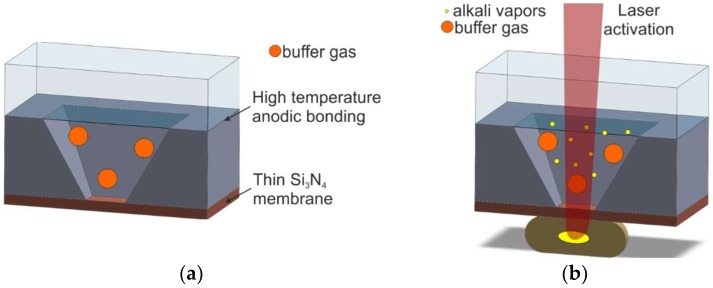
Laser ablation of the silicon nitride membrane and alkali metal introduction: (**a**) cross-sectional view of the MEMS structure, (**b**) laser-induced cesium introduction.

The other method of fabrication of MEMS cesium cells is shown in Reference [51]. First, a micromachined silicon–glass preform is manufactured, with one side covered by a thin silicon nitride layer and filled with a desired buffer gas atmosphere (Figure 5a). After that, a wax container, with a small amount of pure alkali metal sealed inside, is attached to the preform (Figure 5b). In the “hot stage” of cell fabrication, 355-nm laser light ablates the silicon nitride’s thin membrane and evaporates the liquid cesium, forming a gaseous atomic cesium atmosphere inside the cell.

This method may not be applicable industrially due to the easy permeation of the external atmosphere through wax into the cell, but the proposed technique is a very good illustration of creative inventions at the laboratory level.

### 2.2. Chemical Compound Reactions

#### 2.2.1. On-Chip Chemical Reactions of Alkali Metal Chloride and Barium Azide

Several serious negative features of low-temperature anodic bonding to seal cells filled with liquid alkali metal, as well as difficulty accommodating the glassblowing technique in MEMS technology, turned the attention of researchers toward a new method of cesium/rubidium miniaturized cell fabrication based on the generation of alkali metal vapor inside the sealed MEMS structure by the chemical reaction between alkali metal chloride and barium azide. These substances are stable in the air, making them more convenient to handle. The reaction with rubidium chloride can be described as follows [45,52]:

(1a) $${\mathrm{BaN}}_{6}\overset{200\;{}^{\circ}C}{\to }\mathrm{Ba}+3N_{2}↑$$

(1b) $$2\mathrm{RbCl}+\mathrm{Ba}\overset{250-300\;{}^{\circ}C}{\to }2\mathrm{Rb}↑+{\mathrm{RbCl}}_{2}$$

The reaction with cesium chloride, by analogy, is as follows [44]:

(2) $${\mathrm{BaN}}_{6}+2\mathrm{CsCl}\to {\mathrm{BaCl}}_{2}+3N_{2}↑+2\mathrm{Cs}↑$$

**Figure 6:**
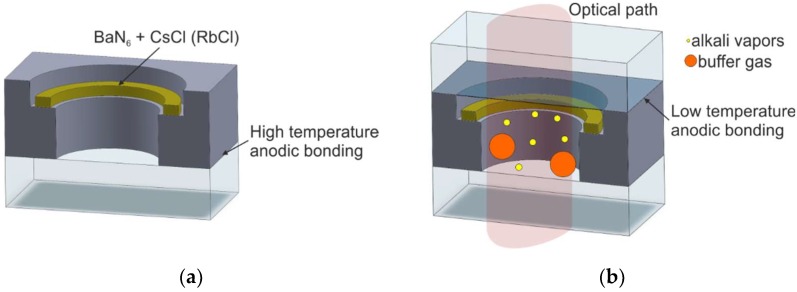
Fabrication process utilizing on-chip chemical reaction: (**a**) silicon–glass preform with BaN_6_ + CsCl (RbCl) ring, (**b**) cross section of the cell.

The utilization of the chemical reaction as alkali vapor introduction allows for significant improvements of the fabrication of MEMS Cs/Rb cells [45,53]. The process consists of the fabrication of a glass–silicon preform, placing a mixture of alkali chloride (CsCl or RbCl) and barium azide inside the preform, and final encapsulation in the buffer gas atmosphere (Figure 6).

The final encapsulation step comprises three phases. Phase one is preheating at 120 °C, which induces a reaction in which barium azide decomposes into barium and nitrogen (nitrogen is then pumped out). After that, a top glass cover is placed onto the silicon–glass preform, a polarizing voltage is applied, and the temperature is elevated to 300 °C. During the thermal ramp, silicon–glass bond is formed, and the second stage of the chemical reaction is done.

This process seems suitable for mass-scale production of MEMS cesium/rubidium cells. However, alkali chloride may be redeposited onto the inner surfaces of the cell, significantly influencing its transparency. The presence of chlorides can act as a buffer gas, among others buffer gases, but this effect has not yet been discussed.

#### 2.2.2. Off-Chip Chemical Reaction of Alkali Metal Chloride and Barium Azide

**Figure 7:**
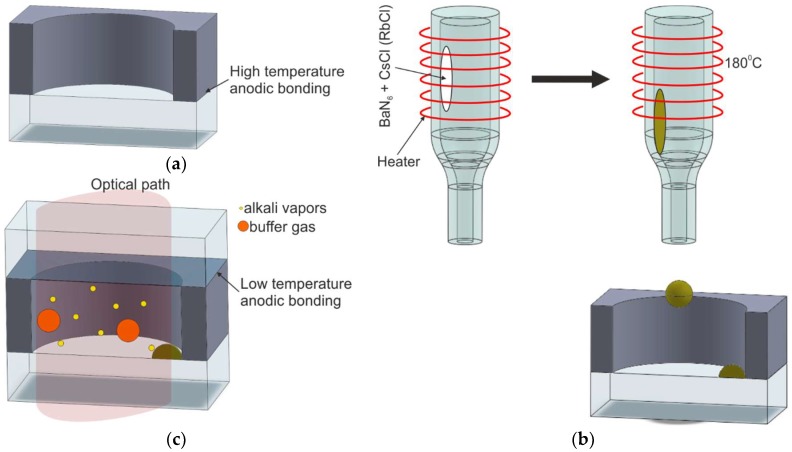
Introduction of cesium into the cell preform by pipetting of pure alkali metal: (**a**) silicon–glass preform, (**b**) drop insertion of liquid alkali metal, (**c**) cross section of the MEMS structure.

The modified method, which combines chemical reaction for the generation of alkali vapors and their direct introduction into the MEMS preform, has been presented by Knappe et al. in Reference [54]. The method utilizes the chemical exchange reaction forming of liquid cesium or rubidium in the form of small drops outside the preform, followed by the low-temperature anodic bonding procedure of the final encapsulation of the cell (Figure 7).

The first step consists of baking the silicon–glass preform and the glass cover at 300 °C inside a vacuum chamber (P = 10^−4^ Pa) in order to eliminate water vapor, which could react with the alkali metal. In the next step, a 30 × 4 mm^2^ glass ampoule with a nozzle that is 700 µm in diameter is used. The ampoule is filled with a mixture of barium azide and alkali chloride. The reaction between the two substances is induced by heating them to 180 °C (heater surrounds the ampoule). As a result of reaction (1), a small “ball” of pure liquid cesium/rubidium is formed at the end of the nozzle. Afterward, the nozzle is placed inside the preform cavity to insert drops of liquid Cs/Rb.

Finally, after the nitrogen has been pumped out (nitrogen also being a product of the reaction) and the chamber has been filled with the appropriate mixture of buffer gases, the preform is sealed by low-temperature anodic bonding of the glass cover.

The method described in this paragraph has been implemented in the fabrication process of glass-blown spherical microcells [55]. The cells have been developed for a nuclear magnetic resonance (NMR) gyroscope [56] or other three-dimensional MEMS.

#### 2.2.3. UV-Induced Chemical Reaction of Alkali Azide On-Chip

**Figure 8:**
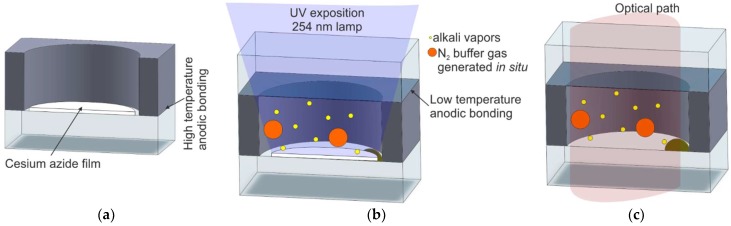
Process steps for obtaining cesium vapor cell using alkali azide deposition and ultraviolet (UV) photolysis: (**a**) silicon–glass preform with deposited alkali azide film, (**b**) UV exposition toward chemical reaction, (**c**) cross section of the cell.

Another method applies the use of the physical deposition process to deposit a layer of alkali azide [45,57,58,59,60] or pipetting of water solution of alkali azide [61,62,63] on the inner side of the cell window. Compound decomposition is induced by exposing encapsulated cells to ultraviolet (UV) radiation. As a result, metallic cesium and nitrogen are obtained inside the cell. The steps of the process are shown in Figure 8.

The procedure consists of compound deposition through a shadow mask, sealed via anodic bonding and UV exposition. The UV radiation is provided by a 254-nm lamp. Several exposition time periods have been tested, ranging from 8 to 100 h. Despite long activation periods, some amount of the alkaline compound was left unreacted inside the cells.

### 2.3. Electrolytic Alkali Metal Introduction

**Figure 9:**
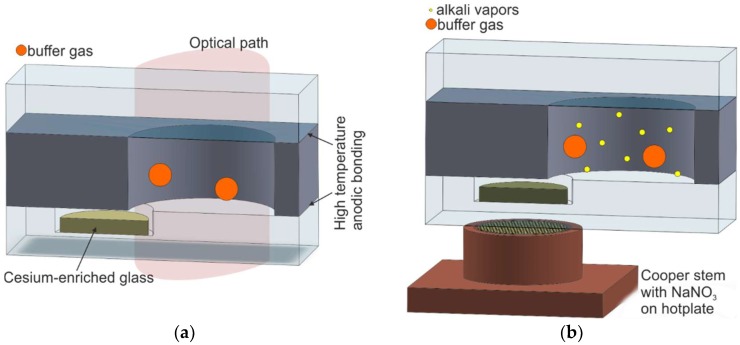
Process of electrolytic alkali metal introduction: (**a**) cross section of the MEMS cell, (**b**) alkali vapor introduction by means of electrolysis.

Another interesting process of cesium MEMS cell fabrication has been proposed in Reference [64]. First, the so-called Cs-glass is formed by melting cesium carbonate and boron oxide at 900 °C and cooling it down to ambient temperatures to form a small, flat piece. The fabrication of the MEMS cell starts from drilling a shallow hole (blind hole) in the glass cover and bonding the substrate to the silicon micromachined body with an intentionally made via hole (Figure 9a). Next, small fragments of the alkali-enriched glass are placed inside the silicon–glass preform, and final encapsulation in the vacuum or another proper gas atmosphere is done. The bonding process is made under optimal parameters (500 °C, 1 kV) so that the formed sandwich is hermetically sealed, and the unwanted emission of postprocess gases is minimized.

In the next step, the structure is heated by a gas flame in order to melt the alkali-enriched glass and allow good contact with the Pyrex glass.

Following the previous step, the cell is placed on top of the copper stem with melted NaNO_3_ (melting temperature of about 300 °C), and the stem is placed on a hotplate heated to 540 °C. When the alkali-enriched glass is positioned directly above the reservoir and the temperature is stabilized, high voltage (700 V) is applied across the structure, initiating the electrolytic current flow. Under such conditions, ionic Na^+^ current is induced. Na^+^ ions flow toward the cesium-enriched glass and replace the Cs^+^ ions. As the end result of this procedure, an atomic cesium vapor is formed inside the MEMS cell.

The invention of this fabrication process has generated a high potential for its functional application in microatomic clocks (Reference [64], published in 2006), but the process has not yet been applied on a wider scale because of the negative features of the method described herein. This includes, among others, cracking as a result of the high mismatch of the thermal expansion coefficient of cesium-enriched glass inside MEMS cell and the Pyrex cover; the formation of ionic channels in the bulk of the bottom glass as a result of sodium ion transportation from NaNO_3_; and possible bond degradation, or even delamination of bonded layers under high-reversed polarization and a large amount of Na^+^ ions generated by electrolysis of NaNO_3_.

### 2.4. MEMS Vapor Cells by Off-Chip Dispensing of Alkali Vapors

#### 2.4.1. Off-Chip Dispensing and Eutectic Bonding

**Figure 10:**
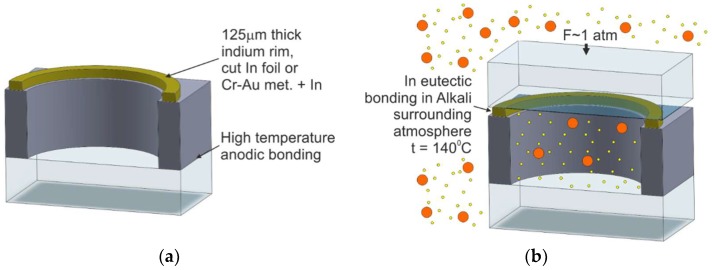
Cell fabrication process utilizing eutectic bonding as the final sealing process: (**a**) silicon–glass preform with Cr–Au met. + In ring, (**b**) cross section of the cell.

MEMS alkali vapor cell technology, utilizing eutectic bonding, has been investigated for years [65,66,67,68,69,70]. According to this technology, the silicon–glass preform and glass cover, kept at a distance, are located in the vacuum chamber. Next, rubidium is evaporated from the liquid phase filling up the inner volume of the chamber and silicon–glass preform. The rubidium vapor condenses inside the preform, forming small, metallic drops of the alkaline metal. This is followed by the thermocompression bonding of the preform and the top glass cover through an indium layer, conducted at 140 °C for 30 min under a pressure of 400 kPa (Figure 10).

This method seems simple and reliable, but vacuum-proof qualities of eutectic sealing of the cell are, at the very least, problematic [71]. There is no data concerning a wider application of the described method in the industry.

The method is relatively simple and repeatable and ensures easy generation of an alkaline-vapor atmosphere, but the Rb vapor generated inside the vacuum chamber makes the surface of the bonded materials passive. Hence, the typical anodic bonding procedure cannot be applied.

#### 2.4.2. Off-Chip Dispensing and Anodic Bonding

**Figure 11:**
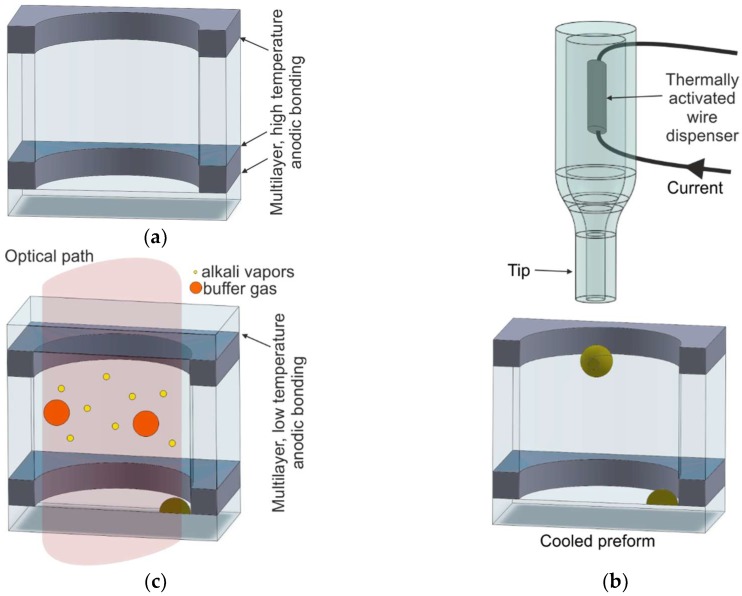
Multilayer cell fabrication: (**a**) four-layer glass–silicon preform, (**b**) drop of rubidium metal inserted into the preform, (**c**) cross section of the MEMS cell.

In this solution [72], the MEMS cell consists of five layers of glass and silicon (Figure 11). First, wafer-scale fabrication of the cell preform is carried out. Two silicon micromachined rims are anodically bonded from both sides to a 3-mm-thick glass wafer with drilled via holes. The first anodic bonding is done under an optimal, high-temperature procedure. The bonded silicon–glass wafer sandwich is then again anodically bonded to a bottom glass cover. In the following step, the glass–silicon/glass–silicon wafer-scale sandwich is sawed into single pieces (Figure 11a), i.e., preforms of cells.

The introduction of rubidium and the final structure encapsulation is done chip-by-chip. The single preform is placed inside a vacuum chamber. A small drop of rubidium metal is delivered by a specialized tool with a wire Rb dispenser into a cooled preform (Figure 11b). Rubidium dispensed from the wire dispenser condenses at the end of the tip, forming a small portion of liquid metal, which is finally inserted inside the preform by an atomic jet. The preform is cooled down, which causes rapid formation of liquid metal, a stable form of rubidium. Next, buffer gases are introduced, and a final anodic bonding of the glass top cover is done at a temperature lower than 300 °C (Figure 11c).

According to the process described in Reference [72], a low-temperature anodic bonding of the final encapsulation procedure is performed without an additional interlayer polarizing electrode. The authors have stated that polarization is placed only at the bottom and top glasses (ion current flows through all wafers of the sandwich). This is probably the biggest drawback of the technology described herein. As is common knowledge [47], the so-called reversed anodic bonding, which mostly occurs in this procedure, influences the sealing quality of all layers of the cell. The possible destruction of the silicon–glass bond is caused by the reduction of Na^+^ ions to Na and NaOH, which can cause irreversible damage to the structure. This may involve problems with the long-term stability of the seal and may significantly increase the permeation of the gaseous external atmosphere and degassing from the bonded interfaces. All of this may influence the long-term run of the cells.

Rubidium/cesium vapors can passivate the surfaces in order to be bonded. The method of rubidium delivery by jet is interesting but raises doubts about the purity of surfaces intended for the anodic bonding.

### 2.5. MEMS Vapor Cells by On-Chip Dispensing of Alkali Vapors

**Figure 12:**
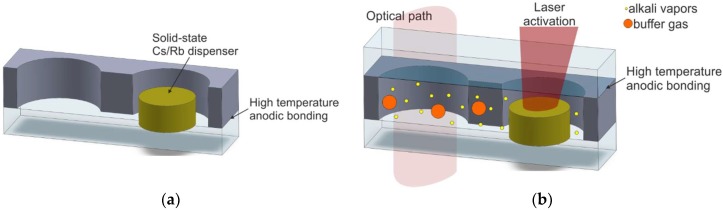
Cell fabrication utilizing solid-state dispenser: (**a**) silicon–glass preform with the dispenser, (**b**) laser-induced on-chip dispensing of alkali vapor.

The most promising MEMS cell technology is based on laser-induced alkali metal vapor dispensing done inside the sealed cells [73,74,75,76,77,78]. The MEMS cell consists of a micromachined silicon body, which has both sides covered with anodically bonded glass wafers (borosilicate glass). The cell contains an optical chamber, a connection channel, and a technical container for a small, pill-like solid-state cesium dispenser from SAES Getters (Milano, Italy) (Figure 12a).

**Figure 13:**
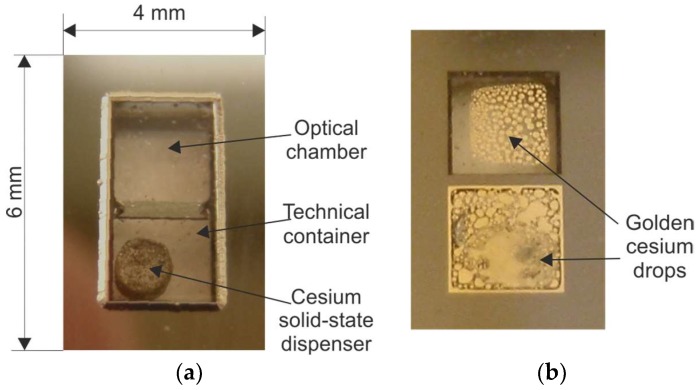
MEMS cell with solid-state dispenser: (**a**) before dispenser activation, (**b**) after the activation.

First, silicon wafers with wet/dry-etched structures are anodically bonded to bottom glass wafers, forming the MEMS preforms. This process is performed using optimal anodic bonding. Next, the dispenser is placed inside the container, and the glass cover is anodically bonded at approximately 400 °C in a buffer-gas atmosphere. After that, atomic cesium is dispensed by heating the solid-state dispenser using a near-infrared spectroscopy (NIR) laser. The dispensing is done cell-by-cell. The laser light spot (980 nm wavelength) is only focused on the dispenser (Figure 12b). It becomes hot while the rest of the cell remains cold. The temperature jumps rapidly from ambient temperature to approximately 720 °C. The high temperature allows a chemical reaction (reduction) between alkali metal salt and reducing agents, such as the metals and metalloids Si, Al, Zr, W, and Fe [79]. The intensity and quantity of alkali atom dispensing can be set with the NIR laser power and irradiation time. Laser irradiation can be performed several times, but it must be done precisely. The process done with the use of absolute minimum laser power and/or for an inadequate length of time will fail because the evaporated Cs/Rb reacts with residuals and passive inner surfaces. Too much laser power and/or a process that is too lengthy will cause the emission of a high quantity of Cs/Rb atoms, which will condense in the form of metal–liquid golden drops, including the optical chamber (Figure 13).

This method ensures a sufficient quantity of atomic cesium/rubidium, controlled by the duration and irradiation power. However, it may involve several disadvantages, for example, the generation of powders coming from the structure of the dispenser, which is usually made from powders in the form of a cylinder by high-pressure compression. This can be minimized by an improved design consisting of several connection channels between the technical container and the optical chamber (a microstrainer) or by the introduction of a microvalve between the aforementioned parts [80,81], which will be shown later in the article.

The other technical disadvantage of the described method is the need for handling, which involves placing the pill dispenser piece-by-piece. Finally, the laser-induced dispenser activation must be performed dispenser-by-dispenser with great precision, as previously mentioned. To overcome the abovementioned setbacks, the mesh-printing of pastes followed by back-up dispensing has been demonstrated [82].

**Figure 14:**
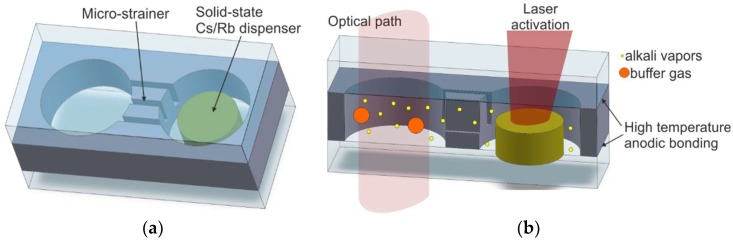
The MEMS cell with microstrainer: (**a**) cell with solid-state dispenser before activation, (**b**) laser-induced on-chip dispenser activation.

An important improvement of the previously described construction is described here. A simple connection channel of optical and dispenser chambers has been replaced by several narrow channels, forming a microstrainer (Figure 14).

The design of the microstrainer needs to solve the issue of finding a compromise between transport efficiency of Cs/Rb atoms and protection against dispenser shards. The problem has been discovered and described in detail in Reference [83].

**Figure 15:**
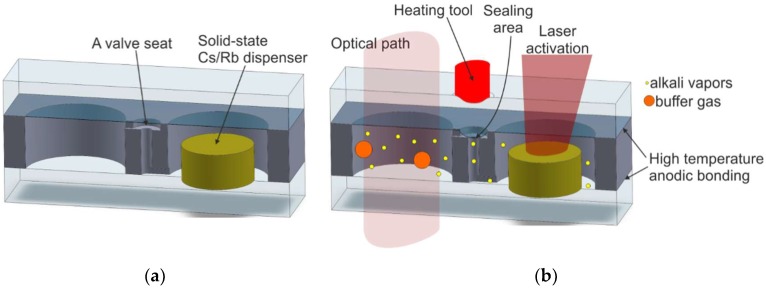
The MEMS cell with microvalve: (**a**) cross section with visible valve seat and solid-state dispenser, (**b**) laser-induced dispensing and closing of the microvalve.

In the next version of the cells based on laser dispensing principle, the new element, a microvalve, has been proposed (Figure 15). A small area of top glass above the valve seat is melted and the valve is closed, separating the dispenser chamber from the optical chamber, in effect protecting it against unwanted contaminations.

The laser dispensing, which creates the alkaline-vapor atmosphere inside both chambers, is followed by temperature treatment of the valve seat (Figure 15b). In this case, subsequent attempts to activate the dispenser are not possible, which is the biggest disadvantage of the solution described herein.

**Figure 16:**
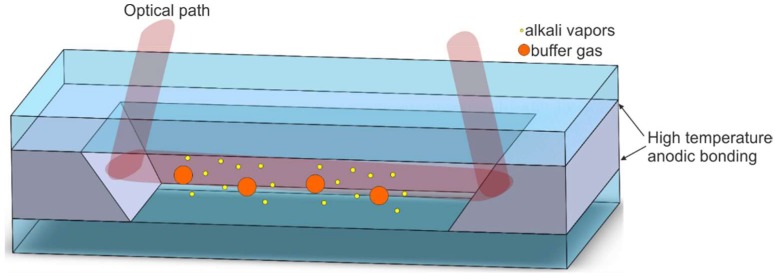
The MEMS cell with reflective sidewalls (pill dispenser, microstrainer, and grating not visible).

Based on the technology described in this paragraph, an alternative cell geometry has been proposed (Figure 16). The solution uses laser beam reflection from the 54.7° silicon sidewalls. This has the advantage that optical path and the interaction of light with atoms is extended. The problem of light reflection from the sidewall laid at a different angle than 45° is solved by proper beam preparation done out of the cell [84,85] or specially designed grating on upper side of the top glass [86], which allows a normal incident of the laser beam.

## 3. Conclusions and Final Remarks

This section gives a comparison of MEMS cell fabrication methods and their applications in microatomic clocks. The results and perspectives on applying the previously described technologies in large scale are commented on as well.

Everything discussed in this paper that has been obtained from literature on the subject of alkali introduction methods and characteristics of cell technology is provided in Table 1.

The positive and negative features of particular methods are described below.

**M1**

Pipetting is a method that ensures effective encapsulation of pure alkali metal and ensures that the buffer gasses are sealed inside the cell. The delivery of pure metal in their liquid form seems to be the simplest solution.

However, a low evaporation temperature and high chemical reactivity of alkali metals must be taken into consideration in the planning of cell technology. Usually, the need for precise liquid manipulation in a vacuum/anaerobic atmosphere makes these techniques difficult to adjust to batch fabrication.

**M2**

The hybrid process of glassblowing and microfabrication was stated by the authors to be a temporary solution. The method was designed to accelerate research in the field of microcell performance while simultaneous work was being done in the field of fabrication processing.

**M3**

The use of metal wax micropackets is very interesting. Unfortunately, postprocessing after the introduction of cesium, detaching the wax containers and the final enclosure of the cell without dehermetizing, has not been reported.

**M4**

The introduction of the alkaline compound appears to be the easiest for large-scale fabrication. The reaction of alkali metal chloride and barium azide is thermally activated. Chemical decomposition of barium azide becomes effective above 120 °C. At room temperature, the substances are stable, even in the air, making them easier to handle. The downsides include the uncertainty of the full conversion of substrates. Moreover, the reaction product (barium chloride) appears as white powder, which can negatively affect the transmission of light through the MEMS cell. A significant fact in this method is that gaseous nitrogen is a by-product of the reaction. The nitrogen can work as a buffer gas, which is considered desirable in some applications.

**M5**

This method uses the advantages of the M4 method and pipetting. The chemical reaction is performed off-chip, reducing problems related to the appearance of solid-state products and unreacted substrates. Pure alkali metal is delivered directly to the silicon–glass preform. Moreover, nitrogen is not required as a buffering gas, which makes it a universal technique with the use of other buffer gases.

**M6**

The method based on UV photolysis of alkali azide deposition applies only well-known microfabrication processes, which are compatible with batch processing. Regrettably, the alkali azide deposition process is not well understood. The deposited films are not uniform, so the amount of alkali metal inside the cell varies from one cell to another. Alkali azide solution dispensing is nevertheless much better controlled. UV activation times using mercury lamps are lengthy (8–100 h), and this process could result in a serious rise in the final product cost. UV laser activation has been demonstrated and shown to have much shorter activation times. Gaseous nitrogen is a product of photolysis. Similar to the M4 description, nitrogen can work as a buffer gas. However, unlike in M4, the amount (pressure) of nitrogen seems to be better controlled.

**M7**

Electrolytic alkali introduction is certainly an unconventional approach. It allows for the precise release of controlled amounts of pure alkali metal inside a sealed cell but, in practice, the realization of a large-scale automatic introduction process would be difficult to arrange. Moreover, the bonding degradation caused by the electrolysis process could lead to long-term instability in the internal gas atmosphere.

**M8, M9**

Off-chip dispensing is an interesting proposition. However, possible passivation of surfaces intended for connection is a possibility. Eutectic bonding does not seem sufficient enough to maintain a proper vacuum level, nor does it maintain the long-term stability of the optical properties of the cells. Multilayer anodic bonding sealing as an alternative sealing process is recommended. Notwithstanding, the running of anodic bonding without an additional intermediate electrode(s) generates negative effects toward long-term quality of the inner atmosphere of the cells.

**M10**

The last presented method, i.e., the use of an alkali dispenser, returned very promising results. It seems that it completely fulfills the requirements stated at the beginning of this chapter. Dispensing is performed after the final sealing of the structure. Anodic bonding is performed at optimal temperatures, which eliminates postprocess problems related to degradation of the bonded interface (degassing). Problems related to the deterioration of transparency of the optical window by fine particles from the dispenser or drops of liquid alkali metal may be eliminated via proper design of the cells.

The latest literature reports indicate that on-chip dispensing from miniature solid-state Cs/Rb dispensers is becoming more and more popular [87,88]. The reason is related to its undeniable advantages. First of all, microdispensers are commercially available as a small pill of ~1 mm in diameter and 0.6 mm height. Activating the dispenser requires the use of an IR laser whose wavelength does not have to be strictly determined. The effectiveness of alkaline vapor release is controlled by laser power and irradiation time. One drawback is the rather large size of the dispenser and the need to manipulate individual dispensers, which can be an obstacle to mass production. However, recent achievements show that the technology has a high applicability in mass-scale production, and the obtained metrological parameters are good [89]. Fractional frequency stability of a CPT-based clock prototype, utilizing Cs vapor cells based on pill dispenser and fabricated as described before, is measured to be 2.5 × 10^−11^ τ^−1/2^ up to 200s averaging time and better than 2 × 10^−11^ τ^−1/2^ at 10^5^ s.

An alternative solution is to use a method based on UV photolysis of alkali azide. A strong improvement of this alkali vapor introduction method has been found recently [61]. The method might be easily scalable to mass production. However, the unavoidable presence of nitrogen as a photolysis product limits the scope of its application.

Among the sealing methods, we can distinguish one dominant method: anodic bonding. This is because this method does not introduce additional materials in contact with highly reactive cesium or rubidium. In new constructions of MEMS cells, the previously used method of connecting through intermediate layers is displaced by anode bonding [71], or new methods are developed using chemically inert materials in relation to alkali metals [88].

The other problem closely related to the subject discussed here is the controlling of the inner atmosphere of MEMS cells. Several publications have shown that residual gases coming from degassing from inner surfaces and interfaces of bonded materials can easily be removed by non-evaporable getters (NEGs), for example, MEMS getters produced by SAES Getters (Italy) [81,90,91]. As well documented in the literature, argon and helium remains ungettered at level of 10^−5^ Torr and 10^−3^ Torr, respectively. This fact limits the ability of using Rb or Cs MEMS cells in miniature atomic clocks based on cooled-atom effects. Here, the vacuum limit must be at least 10^−6^/10^−7^ Torr. In our opinion, the only acceptable solution is to build a MEMS alkali vapor cell in tandem with a micropump. Such a construction was proposed in the literature recently [87,92,93,94] and seems to be a natural step of future solutions.

## Figures and Tables

**Table 1:** Collation of introduction methods.

Method Symbol/Method Description	Reference	MEMS Compatibility	Alkali Vapor Source	Activation Mechanism	Internal Atmosphere Quality	Process Repeatability
M1/Pipetting of pure alkali metal	[15,18,46]	Possible	Pure alkali metal	-	Excellent	Excellent
M2/Hybrid process of glassblowing and microfabrication	[50]	None	Pure alkali metal	-	Excellent	Excellent
M3/Alkali-wax micropackets	[51]	Possible	Evaporation of metallic alkali metal	Laser ablation, λ = 355 nm	Poor	Good
M4/Alkali compound introduction	[45,52,53]	Possible	Reaction between barium azide and alkali chloride	Thermal, T = 200–300 °C	Poor	Poor
M5/Dropping of pure alkali metal obtained by external chemical reaction	[54,55]	Possible	Reaction between barium azide and alkali chloride	Thermal, T = 180 °C	Good	Good
M6/Alkali azide deposition and UV photolysis	[45,57,58,59,60,61,62,63]	Excellent	Photolysis of alkali azide	Long UV lamp activation and shorter UV laser activation	Bad	Bad
M7/Electrolytic	[64]	Probably none	Electrolysis	Electrolytic, T = 540 °C U = 700 V	Poor	Good
M8/Off-chip dispensing and eutectic bonding	[65,67,68,69,70]	Possible	Pure alkali metal or solid-state dispenser	-	Poor	Excellent
M9/Off-chip dispensing and multilayer anodic bonding	[72]	Possible	Pure alkali metal or solid-state dispenser	-	Good	Excellent
M10/On-chip dispensing and high-temperature anodic bonding; structure with microstrainer or microvalve	[73,74,75,76,77,78,80,81,82,84,85,86]	Excellent	Solid-state dispenser	Heating of the dispenser by NIR laser	Excellent	Excellent
